# Solvent-dependent self-assembly of two dimensional layered perovskite (C_6_H_5_CH_2_CH_2_NH_3_)_2_MCl_4_ (M = Cu, Mn) thin films in ambient humidity

**DOI:** 10.1038/s41598-018-23012-2

**Published:** 2018-03-16

**Authors:** Garam Park, In-Hwan Oh, J. M. Sungil Park, Jinyong Jung, Chun-Yeol You, June-Seo Kim, Yonghwan Kim, Jong Hoon Jung, Namjung Hur, Younghak Kim, J.-Y. Kim, Chang Seop Hong, Ki-Yeon Kim

**Affiliations:** 1 Neutron Science Center, Korea Atomic Energy Research Institute, Daejeon, 34057 Republic of Korea; 2 Department of Chemistry, Korea University, Seoul, 02841 Republic of Korea; 3 Department of Emerging Materials Science, DGIST, Daegu, 42988 Republic of Korea; 4 Global Center for Bio-Convergence Spin System, DGIST, Daegu, 42988 Republic of Korea; 5 DGIST Research Center for Emerging Materials, DGIST, Daegu, 42988 Republic of Korea; 6 Department of Physics, Inha University, Incheon, 22212 Republic of Korea; 7 Pohang Accelerator Laboratory, POSTECH, Pohang, 37673 Republic of Korea

## Abstract

Two dimensional layered organic-inorganic halide perovskites offer a wide variety of novel functionality such as solar cell and optoelectronics and magnetism. Self-assembly of these materials using solution process (ex. spin coating) makes crystalline thin films synthesized at ambient environment. However, flexibility of organic layer also poses a structure stability issue in perovskite thin films against environment factors (ex. moisture). In this study, we investigate the effect of solvents and moisture on structure and property in the (C_6_H_5_(CH_2_)_2_NH_3_)_2_(Cu, Mn)Cl_4_ (Cu-PEA, Mn-PEA) perovskite thin films spin-coated on Si wafer using three solvents (H_2_O, MeOH, MeOH + H_2_O). A combination of x-ray diffraction (XRD) and x-ray absorption spectroscopy (XAS) show that relative humidity (RH) has a profound effect on perovskite thin films during sample synthesis and storage, depending on the kind of solvent used. The ones prepared using water (Cu-PEA:H_2_O, Mn-PEA:H_2_O) show quite different behavior from the other cases. According to time-dependent XRD, reversible crystalline-amorphous transition takes place depending on RH in the former cases, whereas the latter cases relatively remain stable. It also turns out from XAS that Mn-PEA thin films prepared with solvents such as MeOH and MeOH + H_2_O are disordered to the depth of about 4 nm from surface.

## Introduction

Since the first report by Kojima *et al*.^1^, we have witnessed that solar cell based on three-dimensional hybrid halide perovskite (ABX_3_, A = CH_3_NH_3_^+^, B = Pb^2+^ or Sn^2+^, X = Cl^−^, Br^−^ or I^−^) as a light absorption and charge separation layer have reached the power conversion efficiency over 20%, comparable to the commercial crystalline Si solar cell technology^1–3^. This scientific breakthrough has been made possible because high quality perovskite structure could be synthesized with low cost and at lower processing temperature via self-assembly using solution processes such as spin coating and dip coating^3–7^. Despite the excellent photovoltaic performance, long term stability of methylammonium based perovskite remains a big challenge to be resolved on the road to practical industrial application. Specifically, it has been reported that crystal structure of three dimensional perovskites become unstable due to the hygroscopic characteristic of amine and power conversion efficiency decays rapidly when they are exposed to humid air environment^8–10^. Extensive efforts have been focused on preventing the structural degradation due to humidity while keeping the high power conversion efficiency^10–15^. These can be summarized with three strategic pathways as follows: (1) tailoring the hole- or electron-transporting layers in solar cell device, (2) protecting the perovskite layer by inserting a hydrophobic passivation layer between the perovskite layer and an upper layer or fully encapsulating a device, (3) making perovskite materials more resistant to humidity by tuning the constituent elements such as organic cation and halide^11–15^. More details on recent overview for improving the long term stability can be found in ref.^16^.

Other than perovskite solar cell, inorganic-organic perovskite thin films have been proposed for a variety of industrial applications such as semiconducting channel in thin film transistors ((C_6_H_5_C_2_H_4_NH_3_)_2_SnI_4_) and multiferroics ((C_6_H_5_C_2_H_4_NH_3_)_2_CuCl_4_ and (C_2_H_5_NH_3_)_2_CuCl_4_) and optoelectronic devices ((C_4_H_9_NH_3_)_2_PbBr_4_)^17–22^. Interestingly, they have two-dimensional layered halide perovskite structure (A_2_BX_4_, A = a monovalent organic cation, B = a divalent metallic cation, X = a halide anion) in common. Bulk perovskites with A_2_BX_4_ formula consist of alternating organic and inorganic layered structure along the one axis. Each octahedron BX_6_ in the inorganic layers comprises six halogen atoms at the vertices and one B atom at the center and they form two dimensional network by sharing corners with each other. Ammonium head group in the organic part has three hydrogen bonds with halogen anions in the inorganic part. Van der Waals interaction between tail groups in the organic layer induces layer by layer self-assembly^17^. It has been reported that organic-inorganic layered perovskite thin films can be readily prepared by a number of simple and versatile techniques such as sol-gel and spin coating and Langmuir-Blodgett, and evaporation^17–22^. It should be noted that the perovskite layers in the previous cases were mostly unencapsulated and exposed to the air even though film thickness ranges from atomically thin to tens of nanometers. However, structure stability of layered perovskite thin film against moisture has been rarely addressed so far. Interestingly, there are reports that two-dimensional homologous perovskite structures with (A)_2_(CH_3_NH_3_)_n−1_B_n_X_3n+1_, where n is an integer, have improved the perovskite’s stability against moisture for solar cell application by partially replacing methylammonium cation with organic cations such as n-butylamine (CH_3_(CH_2_)_3_NH_2_, BA)^23^ or phenylethylamine (C_6_H_5_(CH_2_)_2_NH_3_, PEA)^24^. Nevertheless, layered perovskite thin films are susceptible to moisture from two points of view. Firstly, even BA and PEA are soluble in polar solvents and hygroscopic. Secondly, the surface to volume ratio significantly increases at nanometer scale so surface property could have a profound effect on crystallographic structure of thin films.

**Figure 1:**
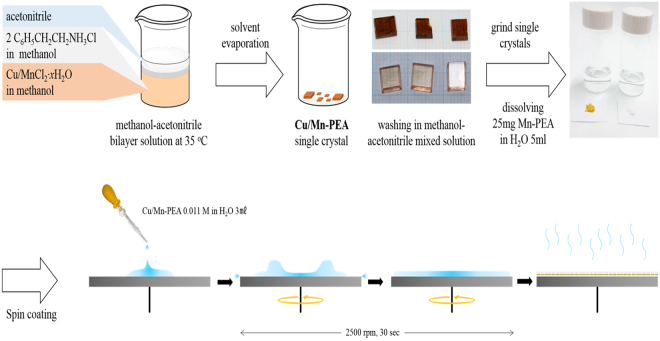
(Schematic procedure showing the synthesis of two dimensional layered organic-inorganic perovskite (C_6_H_5_(CH_2_)_2_NH_3_)_2_(Cu or Mn)Cl_4_ (Cu-PEA, Mn-PEA) thin films using spin coating technique. Note that single crystal was used as a raw material for thin film preparation and perovskite thin films is not encapsulated for surface protection.

In this report, we demonstrate the influence of three different solvents and relative humidity on structural and magnetic properties of unencapsulated two dimensional perovskite films on Si wafer prepared by spin coating technique. To this end, we chose two kinds of layered two-dimensional perovskite films such as (C_6_H_5_(CH_2_)_2_NH_3_)_2_CuCl_4_ (shortly, Cu-PEA) and (C_6_H_5_(CH_2_)_2_NH_3_)_2_MnCl_4_ (shortly, Mn-PEA) on Si wafer without any protection layer by dissolving the good quality single crystals in three different kinds of polar solvents (water, methanol, and a mixed solution of water and methanol) and spin coating them on 4 inch Si wafer, as depicted in Fig. 1. Both Cu-PEA and Mn-PEA belong to a family of layered two dimensional K_2_NiF_4_ perovskites with a chemical formula of A_2_BX_4_ (A = C_6_H_5_(CH_2_)_2_NH_3_^+^, B = Cu^2+^ or Mn^2+^, X = Cl^−^) and inorganic part comprises a two dimensional network of corner-sharing BCl_6_^2−^ octahedron^25^. The interesting point is that these two perovskites crystallize in the same space group (No. 61 P 2_1_/b 2_1_/c 2_1_/a) at room temperature and show the almost same lattice parameters (a = 7.187 Å, b = 7.344 Å, c = 38.549 Å for Cu-PEA, a = 7.207 Å, b = 7.301 Å, c = 39.413 Å for Mn-PEA), but different magnetic behaviors. That is, Cu-PEA is a ferromagnet (F) with Curie temperature at *T*_*c*_ = 9.5~13 K^19,26^ while Mn-PEA is a canted antiferromagnet (c-AF) with Néel temperature at *T*_*N*_ = 43.0~44.3 K^27,28^. According to Goodenough-Kanamori rule, magnetism of A_2_BX_4_ is governed by super-exchange interaction via atomic orbital bridges such as B^2+^ − X − B^2+^ tilting angle, intra- and interlayer B-B distances^29–31^. It was reported that Cu^2+^ in Cu-PEA is Jahn-Teller (JT) active and intralayer ferromagnetism is attributed to alternating *d*_*x*_^2^ − _*z*_^*2*^ and *d*_*y*_^2^ − _*z*_^*2*^ orbital ordering in the octahedron, while Mn^2+^ ion in Mn-PEA is JT nonactive, leading to AF ordering along c-axis and weak F ordering in ab plane due to anisotropic Dzyaloshinsky-Moriya interaction^26,27^.

## Results and Discussion

### X-ray Reflection and Diffraction

**Figure 2:**
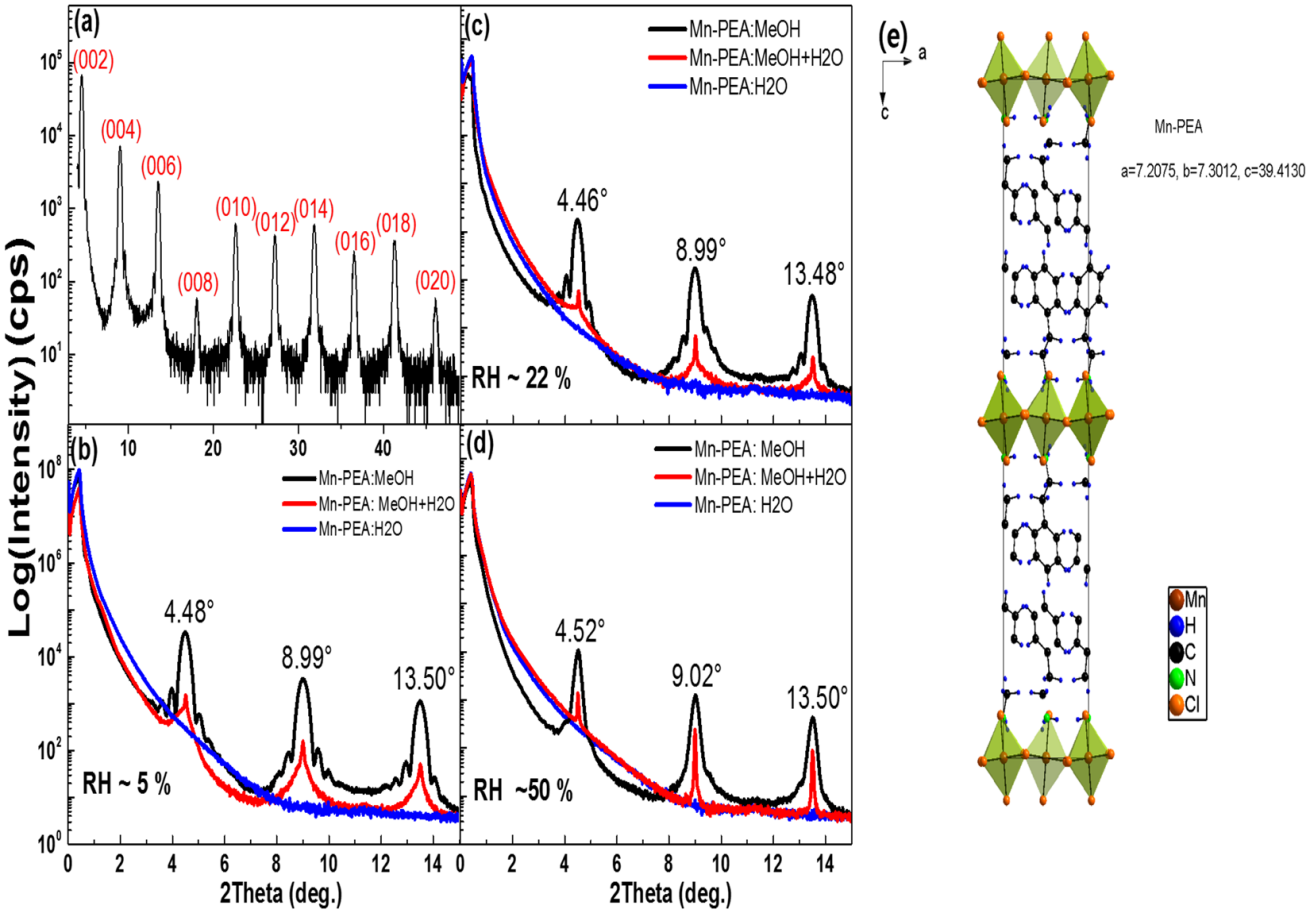
(**a**) Representative x-ray diffraction data of Mn-PEA thin films made with methanol solvent. Low-angle x-ray diffraction data of Mn-PEA:H_2_O, Mn-PEA:MeOH + H_2_O, Mn-PEA:MeOH films spin-cast at three different relative humidity (RH) environment such as (**b**) RH ~ 5% and (**c**) RH ~ 22% and (**d**) RH ~ 50%. (**e**) A figure showing unit cell and lattice constants of Mn-PEA single crystal from the b-axis point of view.

Figure 2a shows characteristic x-ray diffraction (XRD) result of Mn-PEA:MeOH thin films synthesized by spin coating technique. For the sake of convenience, Mn-PEA thin films will be designated hereafter as one of Mn-PEA:H_2_O + MeOH, Mn-PEA:H_2_O, Mn-PEA:MeOH depending on kinds of the solvents, respectively. That’s the same way with Cu-PEA as well. Several (00 $\ell$) diffraction peaks ($\ell$ = 2, 4, 6, …., 20) are clearly observed, indicating that the c-axis of Mn-PEA thin films is oriented parallel to the direction normal to the substrate surface. Out-of-plane distance determined from peak positions is found to be 39.5 ± 0.1 Å. This means that Mn-PEA thin films have the same crystalline structure as bulk single crystal Mn-PEA, as displayed in Fig. 2e. Figure 2b,d demonstrate low-angle x-ray diffraction data of spin-cast Mn-PEA films at three different relative humidity (RH = 5%, 22%, 50%). It is evident that Mn-PEA thin films made with MeOH solvent have a better crystallographic structure than those made with a mixed solvent of MeOH and H_2_O, irrespective of RH. In particular, every diffraction peaks in the Mn-PEA:MeOH thin films (RH = 5%, 22%) are accompanied by high order diffraction peaks at each shoulders. It indicates that unit cells are coherently stacked along the thickness direction. On the other hand, any x-ray diffraction peak is not observed at all in case of Mn-PEA:H_2_O samples and intensity is strongly reduced in case of Mn-PEA:MeOH + H_2_O ones over the experimental RH range. Similarly, x-ray diffraction results of Cu-PEA thin films are also found to show (00 $\ell$) reflection peaks ($\ell$ = 2, 4, 6, …., even numbers) at the same peak positions as bulk Cu-PEA (See Supplementary Fig. S1). The main difference between Mn-PEA and Cu-PEA is that Cu-PEA:H_2_O samples synthesized at RH environment higher than 10% exhibit small but non-zero XRD peaks right after synthesis (See Supplementary Fig. S2). It can be understood that Mn-PEA:H_2_O thin films seem to be more susceptible to moisture than Cu-PEA:H_2_O thin films. In addition, XRD peak intensity and peak shape of Cu-PEA: MeOH + H_2_O are superior to those of Mn-PEA:MeOH + H_2_O at the same RH condition. It is expected that low angle x-ray reflectivity of Cu-PEA and Mn-PEA thin films could measure the total film thickness of organic-inorganic perovskite layer, considering the x-ray scattering length densities of Si (2.01 × 10^−5^ Å^−2^), SiO_2_ (1.88 × 10^−5^ Å^−2^), Mn-PEA(1.00 × 10^−5^ Å^−2^), and Cu-PEA (1.26 × 10^−5^ Å^−2^). However, Kiessig fringe resulting from total film thickness has not been observed. Alternatively, the layer thickness of Cu-PEA thin film was found to be about 250 Å from HR-TEM (Supplementary Fig. S3). Therefore, all the Cu-PEA and Mn-PEA film thickness should be similar with each other because the spin coating conditions (ex. rpm and solvent concentration and solvent volume) were the same.

### Scanning Electron Microscopy

**Figure 3:**
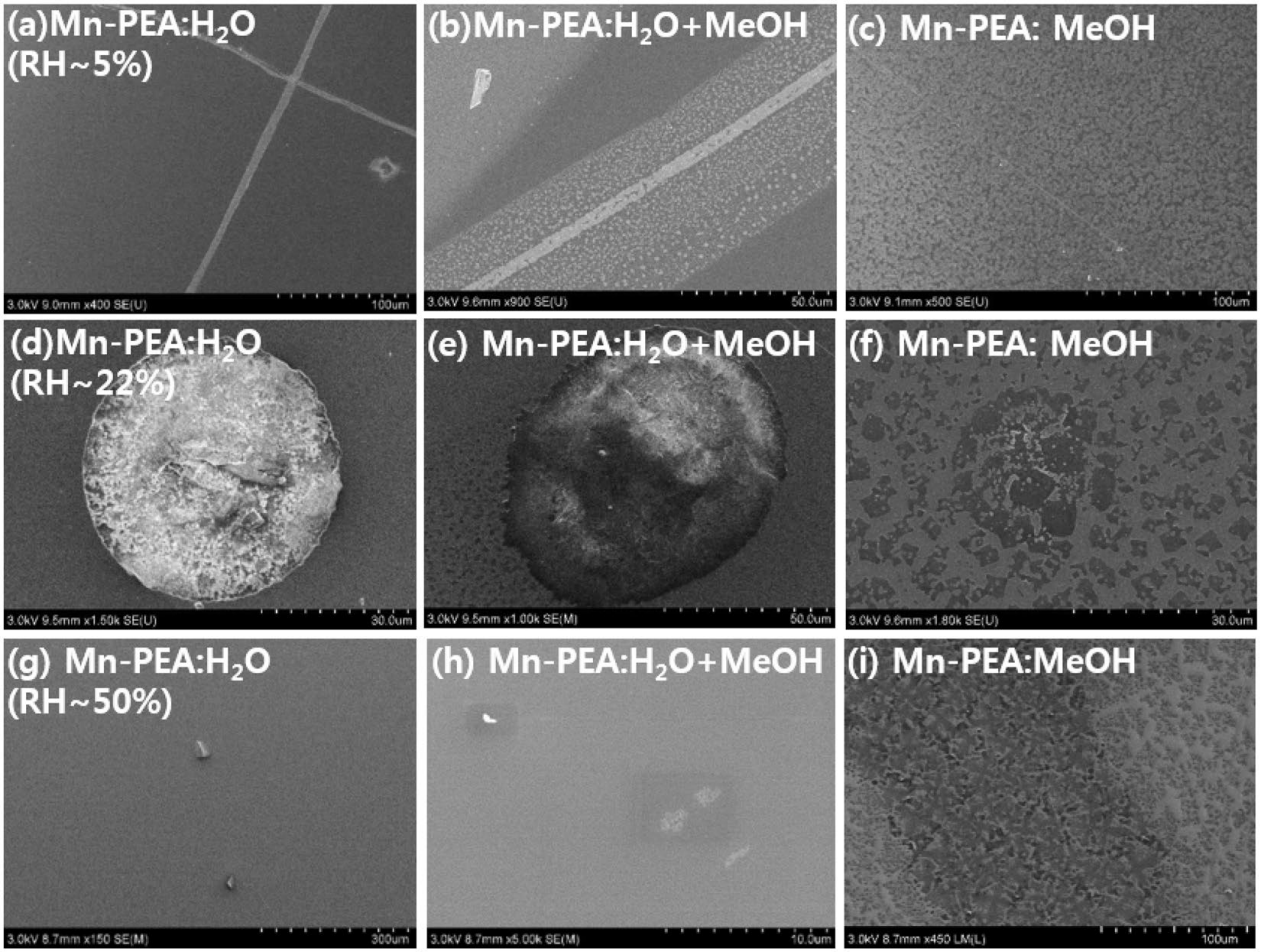
SEM images of three different thin films, that is, Mn-PEA:H_2_O (left), Mn-PEA:MeOH + H_2_O (middle), and Mn-PEA:MeOH (right), depending on RH such as 5% (top), 22% (middle), 50% (bottom). Magnification scale is displayed along with graduated scale at the bottom of each SEM images, which range from 10 μm to 300 μm.

The absence of Kiessig fringe in XRR from total film thickness is found to result from the film morphology. The morphology information such as surface coverage and shape were determined from Scanning Electron Microscopy (SEM) image, as displayed in Fig. 3. It is evident that all the perovskite films are not uniformly coated on Si substrate. The Kiessig fringe in low-angle XRR (Fig. 2b–d) should be averaged out when discontinuous film spin-coated on Si wafer with 4 inch diameter is measured with the incident x-ray beam (square beam, 0.2 mm width × 15.0 mm height). Interestingly, SEM images of Mn-PEA:MeOH samples (Fig. 3c,f and i & Fig. S4c,f,i) have the largest surface coverage and flat thin film structure. On the contrary, the Mn-PEA:H_2_O samples (Fig. 3a,d and g & Fig. S4c,f,i) have so low coverage that most of Si substrate surface looks like uncoated. In case of Mn-PEA:H_2_O (RH~22%, Fig. 3d), tens of micron-sized bubbles containing a number of crystallites can be identified(Supplementary Fig. S4d–f). In addition, single crystals with tens of micrometer size were formed, as shown in Fig. 3g. Considering that all the SEM images of samples prepared at RH~50%(Fig. 3g–i & Fig. S4g–i) were measured at the time of six months after synthesis, not right after synthesis, (Cu or Mn)-PEA:H_2_O samples should end up with single crystals with tens of micron meter or less after enough long time since preparation. We believe that the surface coverage difference between Cu-PEA: H_2_O case and the others is ascribed to the different evaporation rate and wetting properties between water and MeOH organic solvents (Fig. 3 & Supplementary Fig. S5)^32^. This is consistent with the previous report^33^ that surface coverage of spin cast CH_3_NH_3_PbI_3−x_Cl_x_ (x ≪ 1) thin films is reduced as water content dissolved in precursor dimethylformamide (DMF) is increased from 0 to 10%. Therefore, it can be seen that bad morphology in cases of Mn-PEA:H_2_O and Mn-PEA:MeOH + H_2_O is attributed to the fact that water molecules in H_2_O or MeOH + H_2_O solvents have slower evaporation rate and different wetting property, i.e. hydrophobic-like Si substrate surface, compared with organic solvent (MeOH). Although spin coating is the most commonly used technique to grow organic-inorganic perovskite thin film, it seems to be difficult to grow large scale and pin-hole free continuous perovskite thin films. Alternative fabrication techniques such as intercalation method and roll-to-roll slot-die coating and aerosol assisted chemical vapour deposition have been proposed in order to grow the uniform, continuous, and large scale organometal halide perovskite thin films with single crystalline quality for perovskite-based photovoltaics and optoelectronic devices applications^34–38^.

### Temperature-dependent X-ray Diffraction

**Figure 4:**
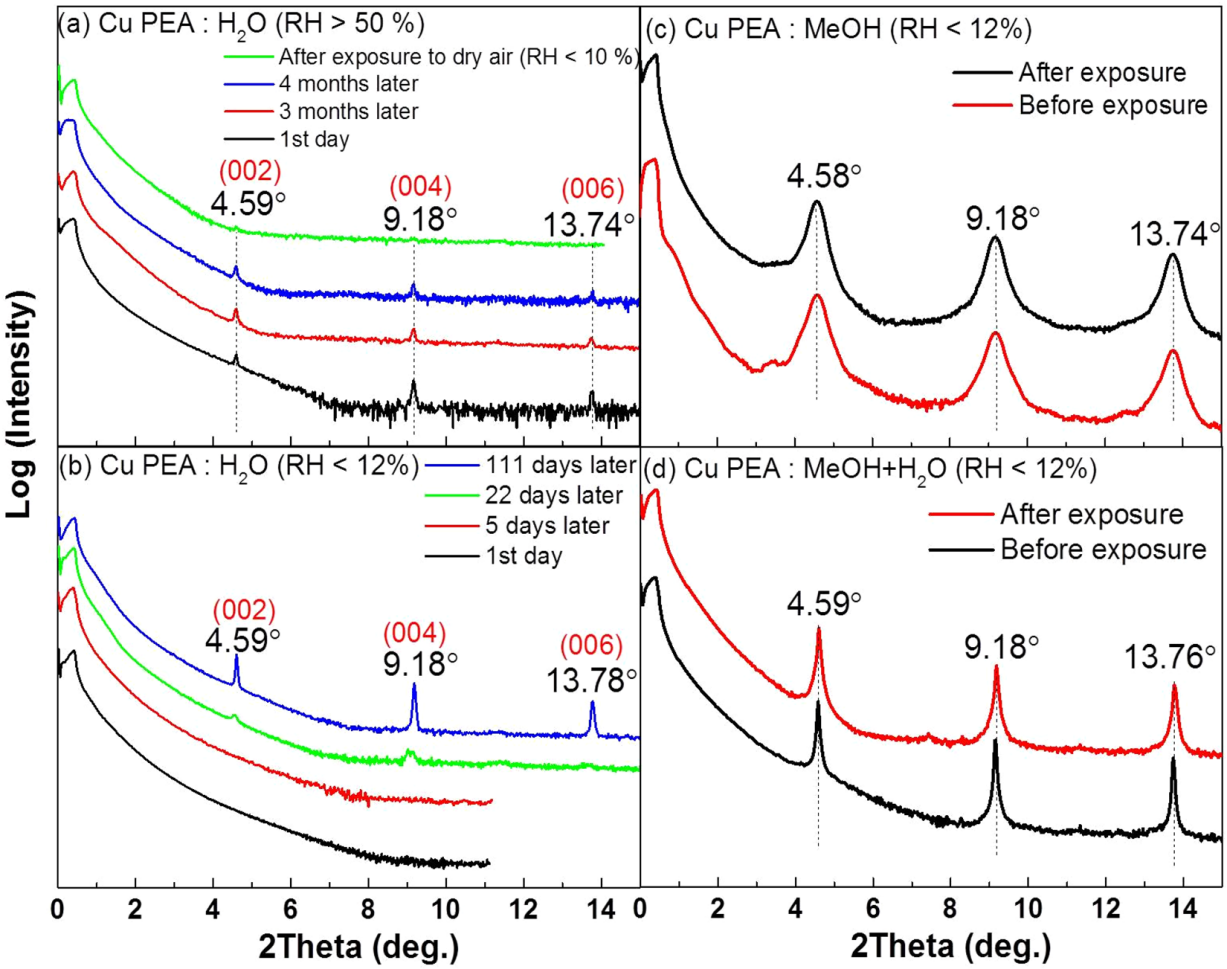
Time-dependent x-ray diffraction analysis of Cu-PEA thin films synthesized made from water solvent (**a**) at high RH over 50% (**b**) at low RH less than 12%. Note that x-ray diffraction measurement was repeated over that time periods that samples had been kept in desiccator with setting of RH less than 30% after synthesis. Cu-PEA thin films made from (**c**) MeOH solvent, (**d**) a mixture of MeOH and H_2_O solvents before and after exposure to dry air (RH < 12%) for a couple of weeks.

Although crystal structures of Cu-PEA and Mn-PEA thin films look very similar to each other irrespective of kind of solvent from x-ray diffraction results, only Cu-PEA and Mn-PEA samples spin-coated with H_2_O solution were found to be very sensitive to RH in the air environment. Figure 4a,b show the low-angle XRD data of the Cu-PEA:H_2_O samples made at two different RH conditions as follows:(1) sample made at RH > 50% for preparation and kept less than 30% inside a desiccator for storage (Fig. 4a) and (2) sample made at RH ~ 12% for preparation and kept less than 30% inside a desiccator for storage (Fig. 4b). When XRD was measured right after Cu-PEA thin film was spin-coated at the very humid condition (>50%), Cu-PEA (00$\ell$) diffraction peaks were clearly observed, as shown in Fig. 4a. As long as Cu-PEA:H_2_O thin films were kept in the RH-controlled desiccator, x-ray diffraction data remain intact after four months. However, XRD patterns completely disappeared after Cu-PEA:H_2_O thin films were exposed to the dry air (RH < 10%) for a couple of weeks. This should result from the evaporation of H_2_O molecules in thin films. On the contrary, the Cu-PEA:H_2_O sample didn’t reveal any XRD peak when spin-coated in a relatively very low RH environment (<12%), as revealed in Fig. 4b. Surprisingly, Cu-PEA (00$\ell$) diffraction peaks were confirmed to appear at about three weeks since samples had been kept in a desiccator with RH less than 30%. They must keep self-assembling by absorbing the moisture from the air over time in desiccators. This process is considered as the moisture-assisted crystallization of Cu-PEA:H_2_O. This structure unstability was found with Mn-PEA:H_2_O thin films as well (Supplementary Fig. S6). It should be pointed out that this transition between amorphous and crystallization state driven by RH in air environment is a reversible process. The Cu-PEA thin films spin-coated with H_2_O + MeOH or MeOH solutions, on the other hand, look relatively stable against the air environment. Even though they were exposed to dry air (RH < 10%) for several days, x-ray diffraction results didn’t change conspicuously, as shown in Fig. 4c,d. Reversible order to disorder transition depending on relative humidity in two-dimensional layered halide perovskite thin films was completely unexpected. Ironically, two dimensional layered lead halide perovskites where MA is partially superseded by PEA for organic layer, were proposed as an attempt to enhance resistance to RH for solar cell application^24^. Although there is no available crystal structure information on Cu-PEA:H_2_O and Mn-PEA:H_2_O at the moment, we believe that they are likely to form hydrated crystal phase, similar to the reversible hydration and dehydration process of CH_3_NH_3_PBI_3_ single crystal and thin films^38,39^.

### X-ray Absorption Spectroscopy

**Figure 5:**
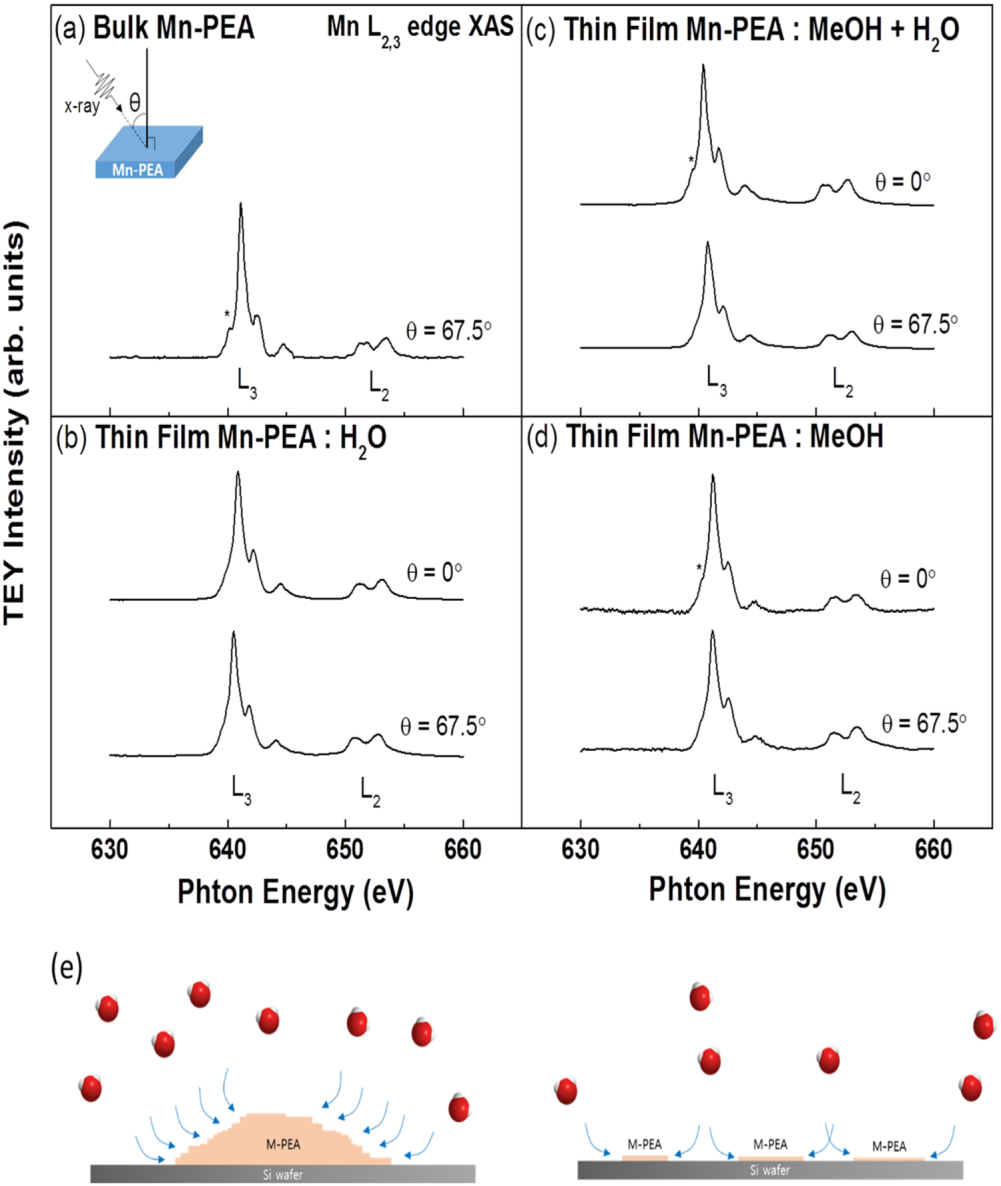
Mn *L*_2,3_ edge absorption spectra taken in TEY mode at temperature lower than 44 K on (**a**) bulk single crystal Mn-PEA, (**b**) Mn-PEA thin film made from water solvent (Mn-PEA:H_2_O), (**c**) Mn-PEA thin film made from a mixed solvent of methanol and water (Mn-PEA:MeOH + H_2_O), (**d**) Mn-PEA thin film made from a methanol solvent (Mn-PEA:MeOH, RH > 50%). The inset figure in (**a**) shows the scattering geometry where two incidence angles (θ = 0°and 67.5°) are adopted. The figure in (**e**) shows how the water molecules in the air permeate into samples having two different growth morphology.

To identify the crystal symmetry of the inorganic perovskite layer in the spin-cast films, we performed x-ray absorption spectroscopy experiment and obtained x-ray absorption spectrum (XAS) at Mn *L*_*2*,*3*_ absorption edges (*L*_*2*_: 649.9 eV, *L*_*3*_: 638.7 eV) in total electron yield (TEY) mode. Bulk Mn-PEA single crystal was also measured as a reference sample for comparison with Mn-PEA thin films. If thin film has the same crystal symmetry as that of the inorganic perovskite layer of bulk single crystal Mn-PEA, Mn *L*_*2*,*3*_ edge XAS from divalent Mn atom with the octahedral crystal environment is expected. Figure 5 reveals the XAS results of Mn-PEA single crystal and thin films measured with linearly polarized x-ray beam at two incident angles (θ = 0°, 67.5°). The incident angle θ is defined as the polar angle between the incident x-ray beam direction and the surface normal. Information on valence, spin states, and covalence of Mn cations octahedrally coordinated by neighboring Cl atoms could be drawn from the comparison with Mn *L* edge XAS of manganese oxide and fluorides such MnO_2_ (Mn^4+^), Mn_2_O_3_(Mn^3+^), MnO(Mn^2+^), MnF_2_(Mn^2+^)^40,41^. Firstly, Fig. 5a shows one peak at leading edge (*L*_*2*_: 651 eV, *L*_*3*_: 640 eV) of *L*_*2*,*3*_ peaks in the Mn *L*_*2*,*3*_ edge XAS of bulk single crystal measured at θ = 67.5°. This must be a direct evidence of a divalent Mn cation in the octahedron coordination environment from the comparison with lineshapes of the previously reported Mn *L*_*2*,*3*_ edge XAS^40,41^. Meanwhile, there is no corresponding peak at leading edge of *L*_*2*,*3*_ peaks for the case of Mn-PEA:H_2_O thin film (RH > 50%) in Fig. 5b. This reveals that a divalent Mn cation has a tetrahedron coordination environment. It strongly suggests that Mn-PEA:H_2_O sample is likely to become disordered due to dry environment, as revealed in Fig. 4. On the other hand, Mn *L*_*3*_ peak lineshape of Mn-PEA: MeOH + H_2_O (RH ~ 5%) and Mn-PEA:MeOH (RH > 50%) thin films are found to retain the octahedral symmetry as well as tetrahedral symmetry when they were measured at θ = 0.0°, revealed by Fig. 5c,d. Given that probing depth at θ = 0.0° is deeper than that of θ = 67.5°, the local crystal structure seems to be disordered to the depth of about 4 nm from the surface due to exposure to air environment. This may be responsible for the unidentable peaks (bet. 10∼12°) other than (00 $\ell$) peaks observed in XRD data of Mn-PEA:MeOH thin films exposed to air. (Supplementary Fig. S7). Mn-PEA: MeOH + H_2_O and Mn-PEA:MeOH thin films are not so susceptible to RH as Mn-PEA:H_2_O thin film. Moreover, XRD results of Mn-PEA: MeOH + H_2_O and Mn-PEA:MeOH thin films remain unchanged even after they were exposed to multiple stimuli such as electric voltage (500 V) and soft x-ray radiation and high temperature (>100 °C) in the presence of oxygen within sample. Secondly, it is known that the spin state in 3d transition metal depends on the relative magnitude between crystal/ligand field effect (Δ) and site-on Hund’s rule exchange (*J*_*H*_, ~2.0 eV)^42^. That is, low spin state when Δ > *J*_*H*,_ high spin state when Δ < *J*_*H*._ It is known as well that high spin state has large branching ratio, *I(L*_*3*_*)/[I(L*_*3*_*)* + *I(L*_*2*_*)]*, between *L*_*2*_ and *L*_3_ peaks in XAS^43^. It is evident that branching ratio in Mn XAS of Mn-PEA single crystal and thin films is larger than those of MnO_2_ and Mn_2_O_3,_ as similar to Fig. 1, ref.^41^. This indicates that Mn^2+^ has high spin state in Mn-PEA thin films as well as bulk crystals. Thirdly, although Mn XAS of MnF_2_ and MnO have the same valence and the same overall lineshape, MnF_2_ shows much sharper *L*_*2*,3_ edges than oxides owing to their ionic character. Similarly, Mn XAS in Mn-PEA samples show much sharper features in both *L*_*2*,*3*_ edges than Mn oxides^41^. Our XAS data clearly demonstrate that Mn atoms have well-localized orbital with ionic character, divalence, and high spin state when they are octahedrally coordinated by chlorines.

Taken together, H_2_O molecules in the environment are likely to permeate into Cu-PEA and Mn-PEA spin cast on Si wafer along the organic pathway, as depicted in Fig. 5e. In addition, It should be unlikely to penetrate the inorganic layers along the thickness direction. Where the water molecules are located in crystal structure needs further research. We suggest that crystal structure and properties of thin film would be resistant against RH if flat, continuous (pinhole-free) thin films could be synthesized. It still remains scientifically challenging to attain the pristine two-dimensional organic-inorganic hybrid perovskites with atomic layer thickness^44^ because water molecules could penetrate the one-unit cell along the direction normal to the surface.

### Temperature-dependent Magnetization

**Figure 6:**
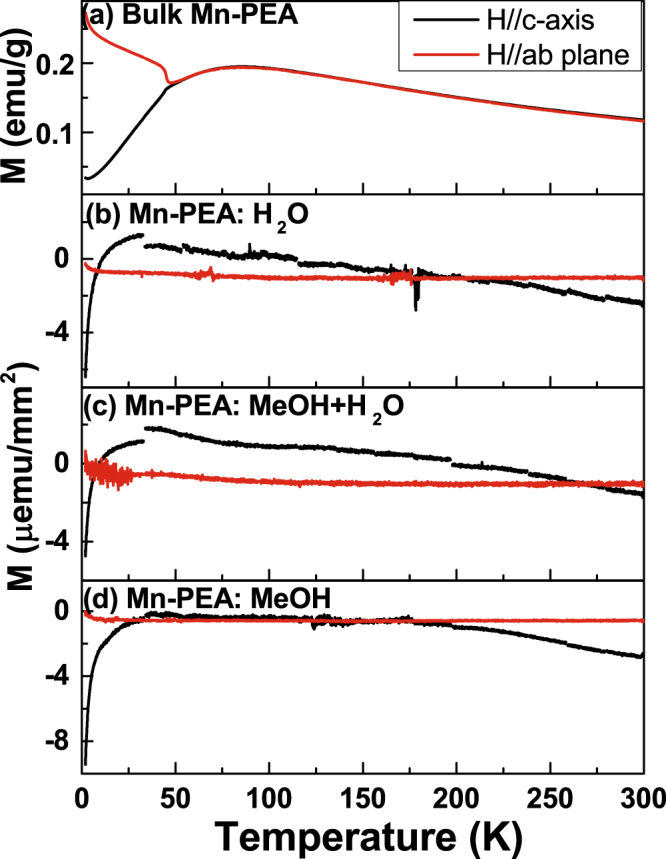
Temperature dependence of magnetization (M-T curve) of (**a**) bulk single crystal Mn-PEA, (**b**) Mn-PEA:H_2_O, (**c**) Mn-PEA:MeOH + H_2_O, and (**d**) Mn-PEA:MeOH thin films. M-T curves were measured for two field orientations where one is parallel to c-axis and the other is parallel to ab-plane. Each was measured under a magnetic field of 0.5 T from 2 K to 300 K after field cooling to 2 K.

To find out the magnetic properties of unencapsulated Cu-PEA and Mn-PEA thin films exposed to air environment, magnetization as a function of temperature from 2 K to 300 K was measured under the presence of a magnetic field less than 1 T along the direction parallel to the film plane and along the direction normal to the film plane using vibrating sample magnetometer option in physical property measurement system. Figure 6a shows that bulk single crystal Mn-PEA is antiferromagnetic below about 45.1 K where *T*_*N*_ is defined as the inflection point in the dM/dT data calculated from M-T curve measured under a magnetic field of 0.5 T along ab-plane. Magnetization of Mn-PEA thin films from M-T curves in Fig. 6b–d has the same order of magnitude (~μemu/mm^2^) as diamagnetic Si substrate (temperture-independent^45^). This leads to the negative value in M-T curves. It can’t be identified if Tc of spin-cast Mn-PEA thin films is below 2 K because transition temperature of hydrated low-dimensional magnets has been known to be greatly reduced^45^. Low magnetic signal to noise ratio makes it difficult to verify whether or not Mn-PEA thin films have the AF phase at low temperature. Nevertheless, Mn-PEA thin films show likelihood of short-range ordering in low-dimensional AF as broad maximum in the temperature dependence of magnetization, as revealed in Fig. 6b–d^46^. Meanwhile, bulk single crystal Cu-PEA is ferromagnetic below *T*_*C*_ = 10 K from M-T curve (see supplementary Fig. S8a). It can be seen that magnetic easy axis is in the ab-plane and c-axis is hard axis. Magnetic hysteresis curves at 2 K for both ab-plane and c-axis (Inset in Fig. S8a) are shown. Interestingly, spin-cast Cu-PEA:H_2_O thin film (Fig. S8b) shows dominant diamagnetic contribution from substrate, whereas Cu-PEA:MeOH thin film (Fig. S8c) shows dominant paramagnetic contribution (inverse temperature dependence^45^) from sample. The reason why Cu-PEA:MeOH film is paramagnet needs to be addressed. According to Mermin-Wagner theorem, the isotropic, finite-range Heisenberg magnetic interactions on one or two dimensional lattices can be neither ferromagnetic nor antiferromagnetic at any finite temperature^47^. However, magnetic ordering in low dimension material can be stabilized if assumptions such as isotropic and finite-range interaction and dimension are not strictly fulfilled. Therefore, Cu-PEA thin film is likely to have long range magnetic ordering because Cu-PEA is known to be the two-dimensional layered ferromagnetic with an easy axis in the film plane^48^. However, it is very tricky to experimentally demonstrate if short- or long-range magnetic ordering exists in the Cu-PEA thin film with one unit cell thick. As our results demonstrate, pristine Cu-PEA thin film is hard to obtain because moisture can give a profound effect on structure if the film surface is exposed to the humid or dry air environment. Recently, self-assembled organic-inorganic MCl_4_^2-^ based perovskite films (M = Cu, Mn, Co) using Langmuir-Blodgett (LB) technique were reported^49,50^. LB technique has several advantages and disadvantages over spin coating technique. Advantages are (1) uniform thin film over large substrate, (2) controlling film thickness layer by layer, and (3) modification of neighboring organic layers. Disadvantages, on the other hand, are (1) not simple and easy to use, (2) expensive and time-consuming, (3) limited to an amphiphilic substances (i.e. a combination of hydrophillic- and hydrophobic-organics) on the water surface. It should be pointed out that water molecules are likely stuck in hydrophilic organic layer sandwiched between neighboring hydrophobic organic layers during sample synthesis process. They have no pathway to exit from film even when exposed to dry environment. Magnetic hysteresis loops in their papers were shown only with very thick films with layer thickness ranging from hundreds of nanometer to over 1 μm. The reason why magnetic moments even in thick films were too weak in their report might be inherent to LB technique. These hydrated phases are well known to significantly reduce the transition temperature of long range ordering although microscopic theory is not available^45^. We hope our results will stimulate further investigation on ultrathin two dimensional perovskite thin films^51^.

## Conclusion

In summary, we investigated the effect of different polar solvents and relative humidity on self-assembled (C_6_H_5_(CH_2_)_2_NH_3_)_2_CuCl_4_ and (C_6_H_5_(CH_2_)_2_NH_3_)_2_MnCl_4_ thin films on top of Si wafer by spin coating technique. X-ray diffraction data of Cu-PEA and Mn-PEA thin films show several (00 $\ell$) reflection peaks ($\ell$ = 2, 4, 6, …., even numbers) at the same peak positions as corresponding bulk materials. Spin coating technique allows us to synthesize the single crystal-like thin films but undesirable shortcoming like nonuniform surface coverage was observed as well from SEM image. Time dependent x-ray diffraction data reveal that thin film crystal structures depend on relative humidity condition at sample synthesis and storage. In particular, Cu-PEA:H_2_O (Mn-PEA:H_2_O) thin films are very sensitive to the relative humidity. Unexpectedly, they exhibit the reversible crystalline(order)-amorphous(disorder) transition depending on relative humidity. That is, x-ray diffraction pattern very slowly emerges over time by absorbing ambient moisture in desiccator which is kept less RH < 30%. X-ray diffraction pattern, on the other hand, completely disappears if they are exposed to dry air (RH ~ 5%) condition for several days. In addition, this transition is found to be reversibly repeated in Mn-PEA:H_2_O thin films. By contrast, thin films produced by solvents such as MeOH and MeOH + H_2_O remain stable even if they are exposed to multiple external stimuli such as moisture and temperature variations (20~373 K) and electric voltage (~500 V) and soft x-ray synchrotron radiation. Further detailed structural information about the inorganic layer in Mn-PEA thin films could be obtained from Mn *L*_*2*,*3*_ edge x-ray absorption spectra. By comparison between our results and previously reported XAS on manganese oxides and fluorides, Mn atoms are found to have divalence, ionic bonding, high spin state when they are octahedrally or tetrahedrally coordinated by halogen ligands. Specifically, XAS of Mn-PEA:H_2_O thin films show that the inorganic layers are alomst composed of tetrahedral Mn^2+^. Meanwhile, XAS of Mn-PEA:MeOH + H_2_O, Mn-PEA:MeOH thin films at x-ray normal incidence reveal that they are made up of a mixture of octahedrally coordinated Mn^2+^ as well as tetrahedrally coordinated Mn^2+^. Although it was difficult to see the magnetic phase transition of Mn-PEA thin films from M-T curve due to low magnetic signal to noise and diamagnetic substrate contribution, Mn-PEA thin films (~20 nm) seem to show the short range ordering in the low dimensional antiferromagnetics as a broad maximum in the temperature dependence of magnetization. Meanwhile, Cu-PEA:H_2_O thin films show only diamagnetic contribution from substrate and Cu-PEA:MeOH thin films are paramagnetic down to 2 K possibly due to hydrated phase.

## Methods

### Thin Film Synthesis

In the first place, phenylethylamine (0.05 mol, 6.06 g, Aldrich) was added in an excess of HCl (37%) dropwise until the reaction stops. At the end of the reaction, C_6_H_5_CH_2_CH_2_NH_3_Cl, a white solid, was formed. It was washed 3 to 4 times with ether and dried in air for one day. After dissolving C_6_H_5_CH_2_CH_2_NH_3_Cl (0.05 mol, 7.88 g, Aldrich) and CuCl_2_^.^2H_2_O (0.025 mol, 3.86 g, Aldrich) or MnCl_2_^.^4H_2_O (0.025 mol, 4.95 g, Aldrich) in methanol (100 ml), two solutions were mixed. Then acetonitrile (100 ml) was slowly added to this solution. Yellow (Cu-PEA)/pale pink (Mn-PEA) color plate single crystals were formed after four days using a slow evaporation method at 35 °C. Cu-PEA/Mn-PEA single crystals were washed in methanol-acetonitrile mixed solvent and dried in air. To prepare the Cu-PEA and Mn-PEA thin films, each Cu-PEA/Mn-PEA powder was prepared by grinding the single crystals as a raw material, respectively. Each Cu-PEA/Mn-PEA powder (25 mg) was dissolved in three different kinds of polar solvents such as H_2_O and methanol (MeOH) and a mixture of methanol-H_2_O (5 ml each, 0.011 M) and spin-cast on Si wafer with one step procedure. That is, 3 ml of each solution was taken, dropped, and spin coated (2500 rpm, 30 sec) onto the Si wafer with native SiO_2_ oxide layer (4 inch in diameter, ShinEtsu, p-type). Contrary to CH_3_NH_3_PbI_3_, annealing process was unnecessary to crystallize Cu-PEA and Mn-PEA thin films. It should be pointed out that thin films were synthesized without a capping layer on top of them in the air environment but keep track of relative humidity (RH) as from sample synthesis.

### Thin Film Characterization

Crystallographic structures of unencapsulated Cu-PEA and Mn-PEA thin films deposited on Si wafer were characterized by x-ray reflectivity and x-ray diffraction (Model: D8 Discover, Bruker-AXS). Film thickness was checked by cross-section high resolution transmission electron microscope (HR-TEM) operating at 300 kV (Model: Titan 80–300 TM, FEI Corp.) at Korea Institute of Science and Technology, South Korea. Sample surface morphology was measured with Scanning Electron Microscopy (SEM, System image resolution 1.0 nm at 15 kV, Model: S-4800, Manufacture: Hitachi), respectively. Magnetic properties such as magnetization as a function of temperature (2 to 300 K) were measured by employing vibrating sample magnetometer option in physical property magnetic system (Model: Quantum Design). They were obtained for both zero field cooling and field cooling cases along the in-plane and out-of-plane directions, respectively. X-ray absorption spectroscopy was measured in the total electron yield (TEY, probing depth ≤ 5 nm) mode with sample drain current at 2 A elliptically polarized undulator beamline of Pohang Light Source, Republic of Korea. Experiment was carried out with Mn-PEA only. Before the introduction of samples into ultrahigh vacuum XAS chamber (<3 × 10^−10^ Torr), they were baked out at 100 °C for 16 hours and cooled down to below 40 K using open closed cycled refrigerator. Electric voltage of 500 V was applied to backside of the sample throughout the experiment to amplify the signal. The X-ray absorption spectra (XAS) of Mn atoms at *L*_*2*_,_3_ edges in three different Mn-PEA thin films along with bulk Mn-PEA for the control sample were measured below magnetic transition temperature (44.3 K) of bulk Mn-PEA to minimize the effect of thermal motion.

## Electronic supplementary material

Supplementary Information
