# Supplementary material for: Solvent-dependent self-assembly of two dimensional layered perovskite (C6H5CH2CH2NH3)2MCl4 (M = Cu, Mn) thin films in ambient humidity
Source: Sci Rep. 2018 Mar 16;8:4661. doi: 10.1038/s41598-018-23012-2 (PMC5856791; doi:10.1038/s41598-018-23012-2)
Supplement: Supplementary file 1 — Supplementary Information [file 41598_2018_23012_MOESM1_ESM.docx]

Supplementary Information

Solvent-dependent self-assembly of two dimensional layered perovskite (C_6_H_5_CH_2_CH_2_NH_3_)_2_MCl_4_ (M= Cu, Mn) thin films in ambient humidity

Garam Park^1,2^, In-Hwan Oh^1^, J.M. Sungil Park^1^, Jinyong Jung^3,4^, Chun-Yeol You^3,4^, June-Seo Kim^4,5^, Yonghwan Kim^6^, Jong Hoon Jung^6^, Namjung Hur^6^, Younghak Kim^7^, J.-Y. Kim^7^, Chang Seop Hong^2^ & Ki-Yeon Kim^1^

*^1^Neutron Science Center, Korea Atomic Energy Research Institute, Daejeon 34057, Republic of Korea*

*^2^Department of Chemistry, Korea University, Seoul 02841, Republic of Korea*

*^3^Department of Emerging Materials Science, DGIST, Daegu 42988, Republic of Korea*

*^4^Global Center for Bio-Convergence Spin System, DGIST, Daegu 42988, Republic of Korea*

*^5^DGIST Research Center for Emerging Materials, DGIST, Daegu 42988, Republic of Korea**^6^Department of Physics, Inha University, Incheon 22212, Republic of Korea
^7^Pohang Accelerator Laboratory, POSTECH, Pohang 37673, Republic of Korea*

Correspondence and requests for materials should be addressed to K.-Y. Kim (e-mail: [kykim3060@kaeri.re.kr](mailto:kykim3060@kaeri.re.kr))

**
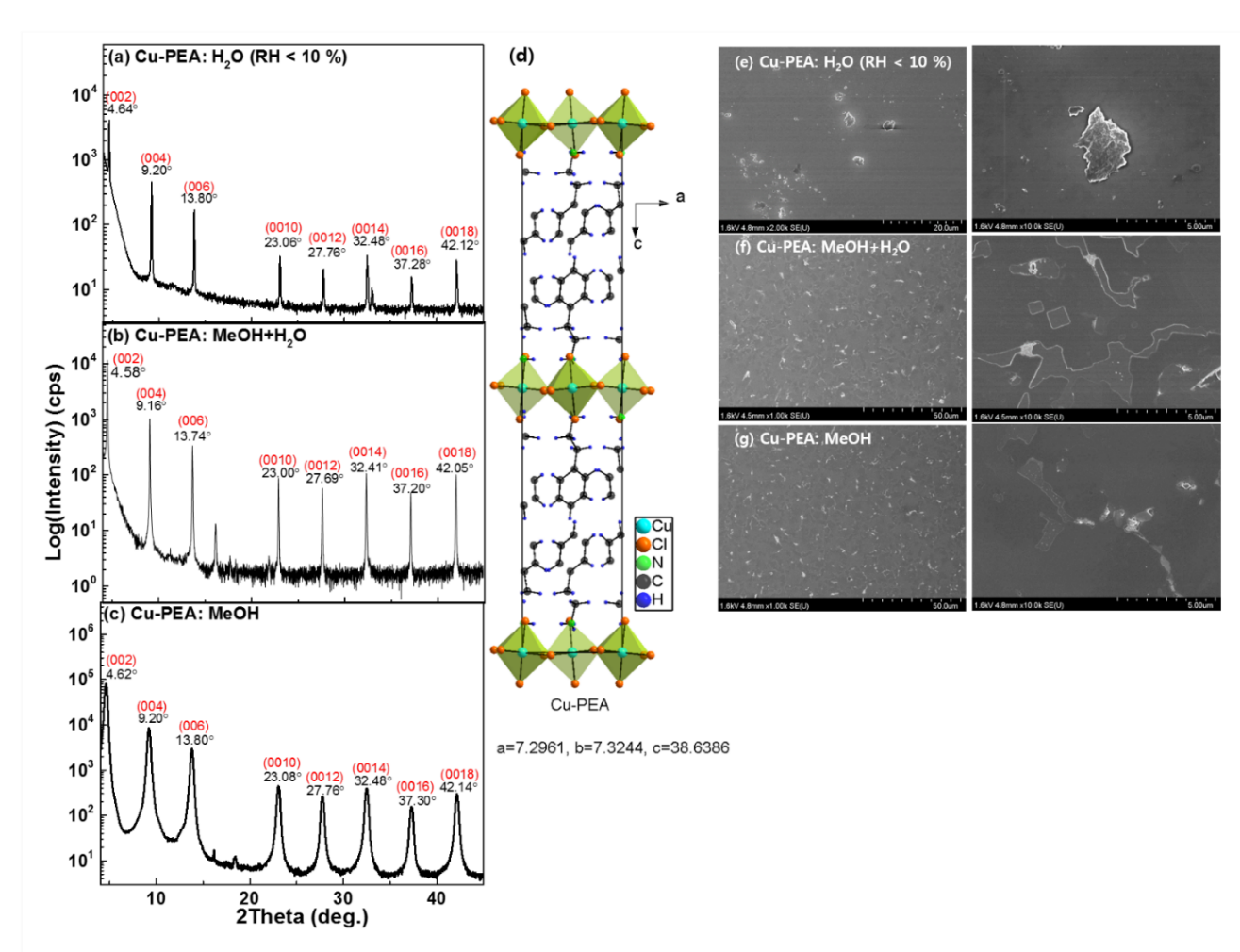
 Figure S1.** X-ray diffraction results of Cu-PEA thin films made from (a) water solvent, (b) a mixture of methanol and water solvent, and (c) methanol solvent. These samples were made at low RH (< 10 %) condition. (d) a figure showing the unit cell structure and lattice parameters of Cu-PEA single crystal, scanning electron microscopy (SEM) images taken with corresponding samples made with (e) Cu-PEA:H2O, (f)Cu-PEA:MeOH+H2O, and (g) Cu-PEA:MeOH solvents

Figure S1 shows x-ray diffraction results of Cu PEA thin films synthesized by spin coating technique. Several (00) reflection peaks (= 2, 4, 6, ...., 18). are clearly observed, indicating that the c-axis of Cu-PEA thin films is oriented parallel to the direction normal to the substrate surface, irrespective of kind of solvents used in spin coating. Out-of-plane distance determined from peak positions is found to be 38.5 ± 0.1 Å. This indicates that Cu-PEA thin films have crystalline structure similar to bulk single crystal, as shown in Fig. S1d. SEM images of Cu-PEA:H_2_O case in Figs. S1e has so low coverage that most of Si substrate surface looks like uncoated, whereas the surface coverage of Cu-PEA:MeOH+H2O and Cu-PEA:MeOH cases look much better.


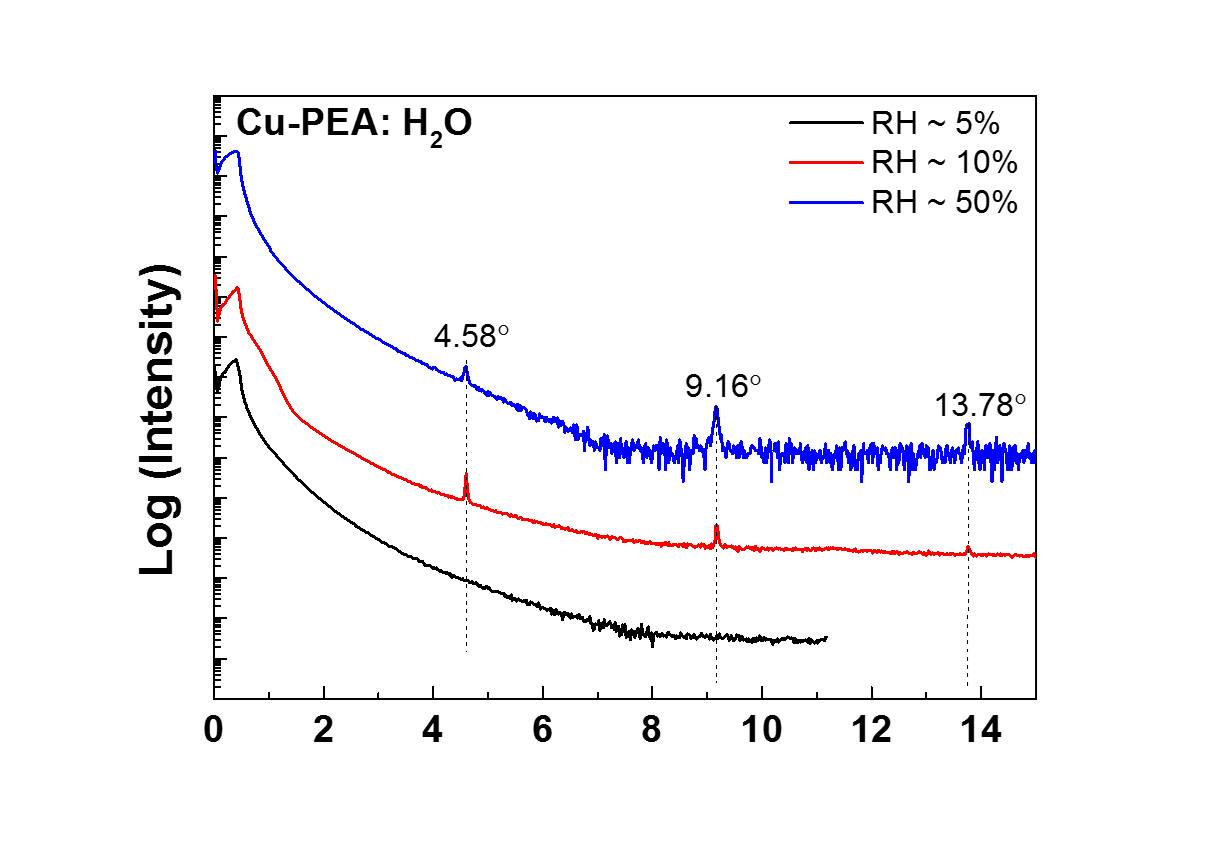


**Figure S2**. Time-dependent x-ray diffraction results of Cu-PEA: H2O samples made at three different RH (~5%, ~10%. ~50%)


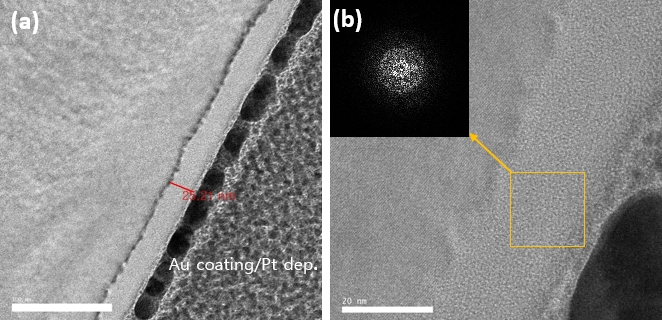
 **Figure S3.** (a) Cross-section transmission electron image and (b) Selected Area Electron Diffraction (SAED) of the Cu-PEA:MeOH+H_2_O thin film deposited on Si water. TEM specimen was prepared by the focused ion beam milling.

Cu-PEA:MeOH+H_2_O thin film thickness synthesized by spin coating were measured with cross-section transmission electron microscopy (TEM) image, as shown in Figure S3 (a). The thickness is found to be about 25 nm. Thickness was confirmed to be similar as well by ellipsometer (Model : MG-1000, Nano-View Co.). In addition, we investigate the local microstructure by Fourier transforming local high resolution TEM images. Figure S3 (b) reveals that it’s amorphous, inconsistent with x-ray results. This should be caused by the focused ion beam milling which degrades the structure of organic-inorganic hybrid films.


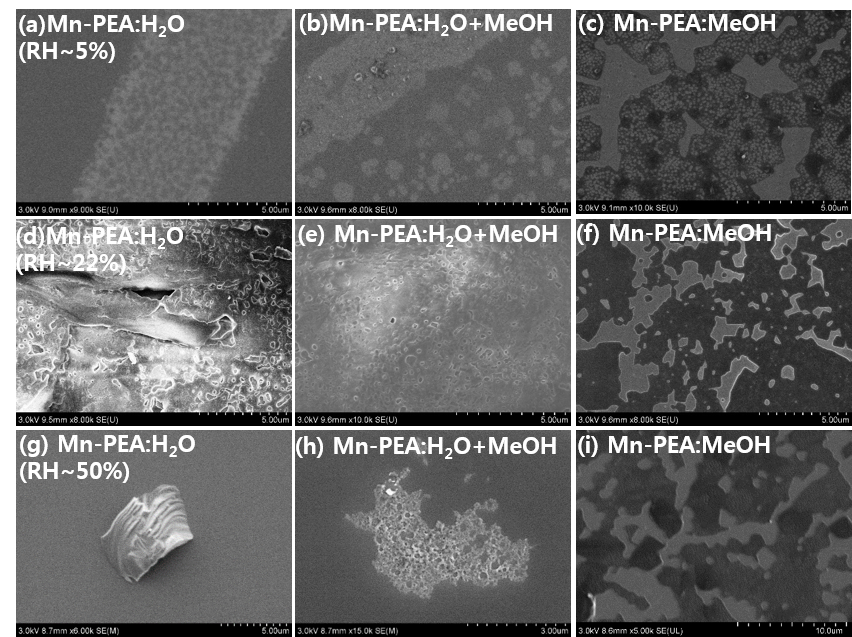


**Figure S4**. SEM images of three different Mn-PEA thin films with magnification ranging from 3 μm to 10 μm. Magnification scale is displayed along with graduated scale at the bottom of each SEM images. RH = 5 % (top), 22 % (middle), 50 % (bottom).


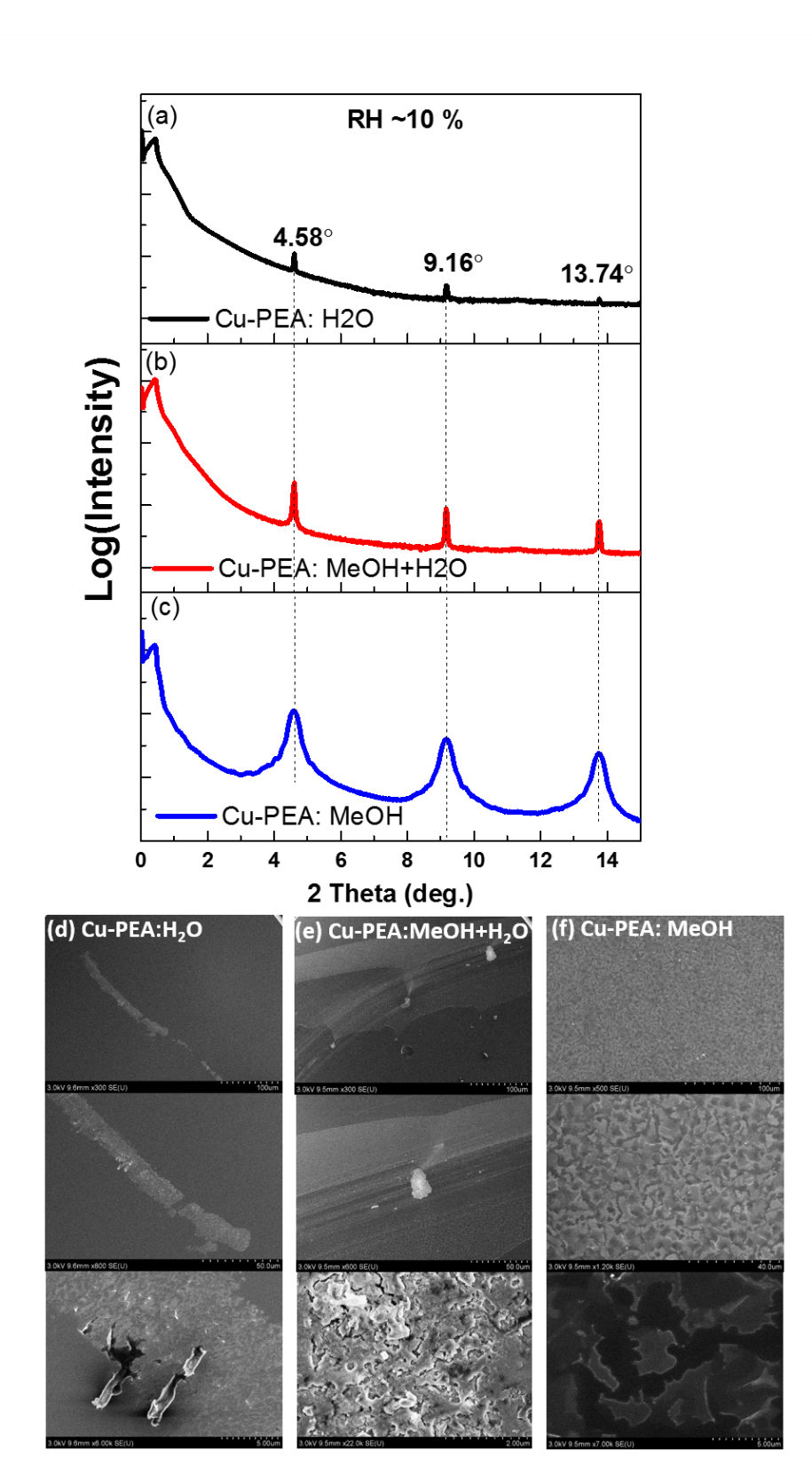


**Figure S5**. X-ray diffraction results (upper figure) of Cu-PEA thin films made from (a) a mixture of methanol and water solvents, (b) methanol solvent, and (c) water solvent. These samples were made at RH (~10 %) condition. Corresponding SEM images of three samples are shown in the lower figure.


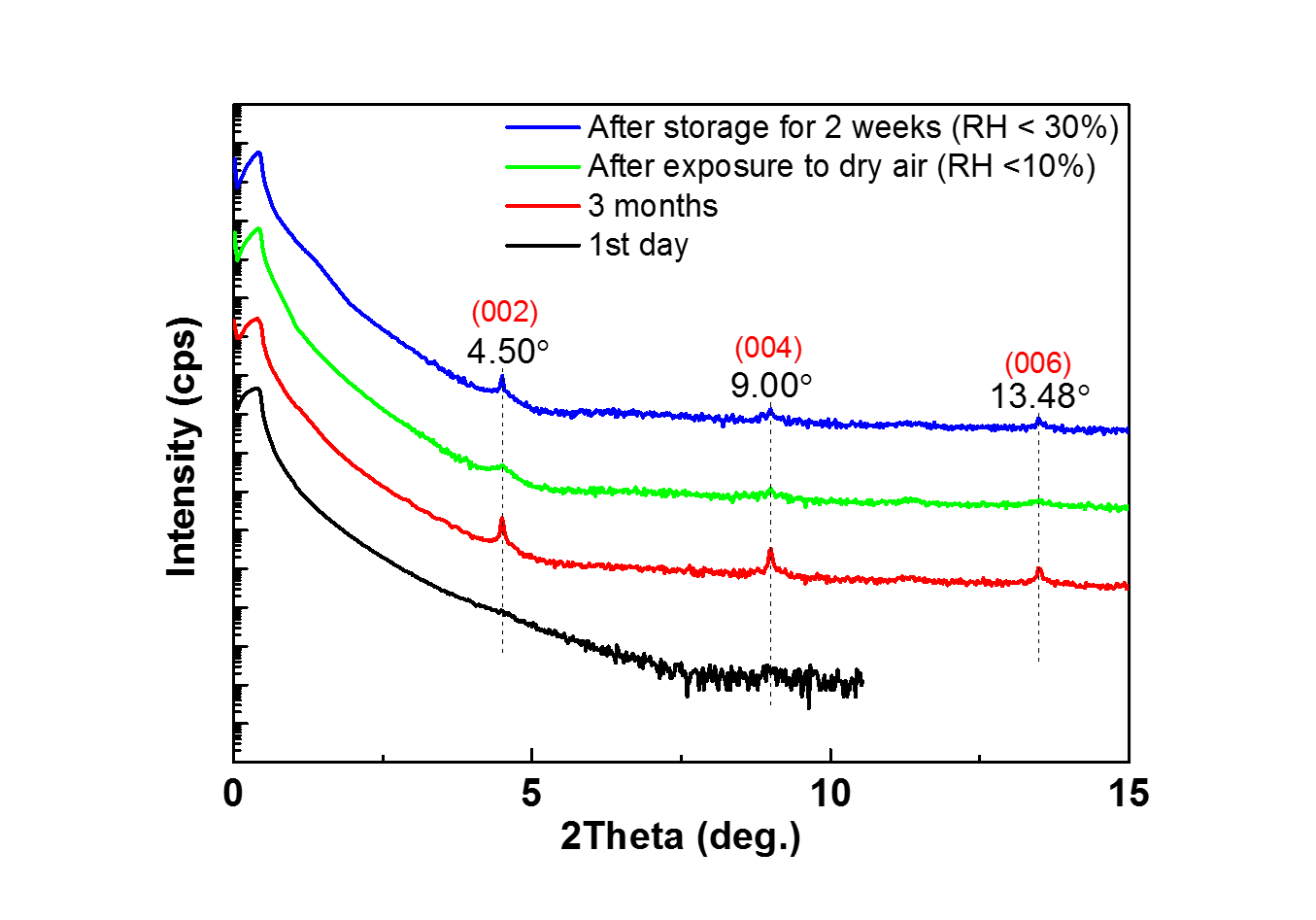
 **Figure S6.** Time-dependent x-ray diffraction analysis of Mn-PEA thin films made from water solvent at high relative humidity (RH ~50 %).

Even Mn-PEA:H_2_O thin films synthesized at high RH(> 50%) environment doesn’t any diffraction peak (a black line in Figure S6) at all when measured right after synthesis. Interestingly enough, x-ray diffraction peaks (a red line in Figure S6) at the same peak positions as bulk Mn-PEA were found to emerge three months since samples are kept in RH(<30%)-controlled desiccator. X-ray diffraction measurement was repeated after exposed to dry ambient environment (RH ~5%) for several days. This leads to the disappearance of diffraction peaks, as depicted as a green line in Figure S6. After two weeks of storage back in desiccator (RH<30%), diffraction peaks were recovering. This is evidence that Mn-PEA:H_2_O thin films undergo the reversible crystallization-amorphous structure phase transition driven by ambient moisture. In addition, Mn-PEA:H_2_O thin films synthesized at low RH(~5%) environment was not recovered even though they were kept in RH-controlled desiccator.

** Figure S7.** X-ray diffraction patterns of Mn-PEA:MeOH thin films (a) measured right after since synthesis (RH~%0%), (b) exposed to ambient humidity for a long time. Other peak except for (00) peaks are designated as color arrows.

**Figure S8.** Temperature dependence of magnetization (M-T curve) of (a) bulk single crystal Cu-PEA, (b) Cu-PEA:H2O, (c) Cu-PEA:MeOH thin films. M-T curves were measured for two field orientations where one is parallel to c-axis and the other is parallel to ab-plane. Each were measured under a magnetic field of 0.01 T from 2 K to 300 K after field cooling to 2 K.
